# Recent Progress of Lipid Nanoparticles-Based Lipophilic Drug Delivery: Focus on Surface Modifications

**DOI:** 10.3390/pharmaceutics15030772

**Published:** 2023-02-26

**Authors:** Yoseph Seo, Hayeon Lim, Hyunjun Park, Jiyun Yu, Jeongyun An, Hah Young Yoo, Taek Lee

**Affiliations:** 1 Department of Chemical Engineering, Kwangwoon University, 20 Kwangwoon-Ro, Nowon-Gu, Seoul 01897, Republic of Korea; 2 Department of Biotechnology, Sangmyung University, 20, Hongjimun 2-Gil, Jongno-Gu, Seoul 03016, Republic of Korea

**Keywords:** lipid nanoparticles, drug delivery systems, lipophilic drugs, solubility, lipid-based colloidal carriers, PEGylation, chitosan coating, surfactant protein

## Abstract

Numerous drugs have emerged to treat various diseases, such as COVID-19, cancer, and protect human health. Approximately 40% of them are lipophilic and are used for treating diseases through various delivery routes, including skin absorption, oral administration, and injection. However, as lipophilic drugs have a low solubility in the human body, drug delivery systems (DDSs) are being actively developed to increase drug bioavailability. Liposomes, micro-sponges, and polymer-based nanoparticles have been proposed as DDS carriers for lipophilic drugs. However, their instability, cytotoxicity, and lack of targeting ability limit their commercialization. Lipid nanoparticles (LNPs) have fewer side effects, excellent biocompatibility, and high physical stability. LNPs are considered efficient vehicles of lipophilic drugs owing to their lipid-based internal structure. In addition, recent LNP studies suggest that the bioavailability of LNP can be increased through surface modifications, such as PEGylation, chitosan, and surfactant protein coating. Thus, their combinations have an abundant utilization potential in the fields of DDSs for carrying lipophilic drugs. In this review, the functions and efficiencies of various types of LNPs and surface modifications developed to optimize lipophilic drug delivery are discussed.

## 1. Introduction

Drug development is essential for treating incurable diseases and prolonging life. Over 40% of new drugs approved for treating various diseases are lipophilic [1]. However, the low solubility of lipophilic agents makes them inefficient for a direct administration in humans [2]. Therefore, the need for a special delivery system to compensate for this is emphasized. Chemotherapeutic drugs directly administered without a delivery vehicle have a short half-life, poor solubility, and severe side effects due to cytotoxicity and a lack of specificity [3]. In addition, drugs at off-target sites reduce treatment efficiency, have low bioavailability, and may cause side effects [4]. A representative example is an mRNA-based COVID-19 (SARS-CoV-2) pandemic vaccine. Although a unique delivery vehicle has been used to deliver the unstable mRNA vaccine into the body [5,6], considerable side effects have been reported in some patients [7,8,9]. This is due to the absent targeting ability of the vaccine, causing an off-target effect, and the carrier comprises some materials that can cause inflammation, including myocarditis [10,11,12,13,14]. Therefore, methods have been devised to deliver more stable, effective, and bioavailable drugs. Over the past several decades, various DDS platforms have been developed to solve these problems. The ongoing DDS studies aim to improve pharmacological efficacy and minimize toxic side effects [15,16].

**Figure 1:**
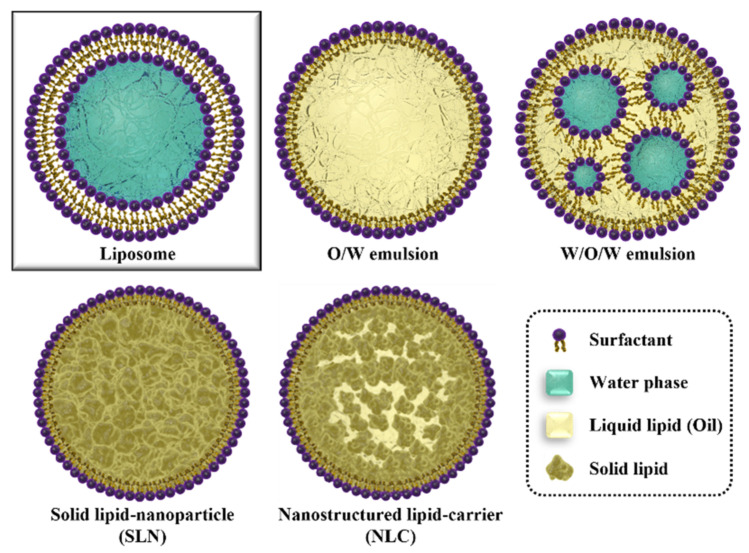
Conventional lipid-based colloidal carriers (liposome and various LNP types).

Among the various DDSs, lipid-based colloidal carriers (LCCs) are biodegradable and non-toxic [17]. Most LCC components are lipids; therefore, LCCs are considered the safest DDS [18]. Liposomes are the most representative LCC and were first described in the 1960s by Alec D Bangham [19]. They have the same phospholipid bilayer structure as the cell membrane (Figure 1). Thus, many researchers have focused on the possibility of treating various diseases using LCCs [20,21]. Numerous studies have been conducted to improve the bioavailability of liposomes, such as drug loading, increased residence time in the body, extrusion to ensure uniform size, and antibody-based targeting [22,23]. These efforts have led to clinical trials in various medical fields, including liposome-based anticancer drugs, antibiotics, gene therapy, and anesthetics [20]. Currently, many liposome-based treatments, such as Doxyl^®^ (doxorubicin), AmBisome^®^ (Amphotericin B), and daunoXome^®^ (daunorubicin), have been approved and used in medicine [24]. However, the high production cost, limited physical stability, low drug-loading capacity, leakage of encapsulated drugs, and complexity and use of toxic organic solvents in the manufacturing process restrict the commercialization of liposomes [25,26]. This situation requires a new alternative; thus, lipid nanoparticles (LNPs) were developed as novel LCCs to overcome the limitations of liposomes [21,25].

LNPs comprise lipids, surfactants, polymers, and emulsions or colloidal nanoparticle structures [27,28]. Unlike liposomes, LNPs mainly consist of phospholipids, a surfactant-based monolayer, and an interior filled with hydrophobic materials (Figure 1) [24]. The synthesis of LNPs is more straightforward to scale up than that of liposomes because they are produced by emulsification between an organic phase and an aqueous phase using the properties of surfactants [29,30]. In addition, they have many advantages, such as a low cytotoxicity, low cost, high stability, and drug-loading efficiency [6]. Previous studies on LNP preparation, characterization, drug loading, and delivery have demonstrated their great potential as a DDS. Generally, drugs are loaded in the carrier during the manufacturing process. Thus, their hydrophobic internal structural characteristics make them suitable for lipophilic drug delivery. Furthermore, because LNPs have a hydrophilic surface that can be well dispersed in aqueous solutions, they have great potential for application as a lipophilic DDS platform in the body (Figure 1) [31].

Since many lipophilic drugs are currently being developed, DDSs to increase the bioavailability of the drug are attracting attention. LNPs can improve the bioavailability of lipophilic drugs owing to their hydrophobic internal and hydrophilic surface structures, excellent biodegradability, and low toxicity, which can overcome the limitations of conventional DDSs. In addition, recent studies have revealed that surface characterization through LNP surface modification can impart high functionality, such as a more precise targeting ability and high-endosome escape to LNP [32,33]. Therefore, many studies have been conducted to modify LNP surfaces to improve lipophilic drug delivery efficiency further [34,35,36]. Here, the various LNPs that deliver lipophilic drugs are considered in this review. In addition, the types and functions of surface modification in LNPs were discussed, and research trends for an efficient drug delivery were explored.

## 2. Various Types of Lipid Nanoparticles

### 2.1. Liquid Lipid-Core LNP

**Figure 2:**
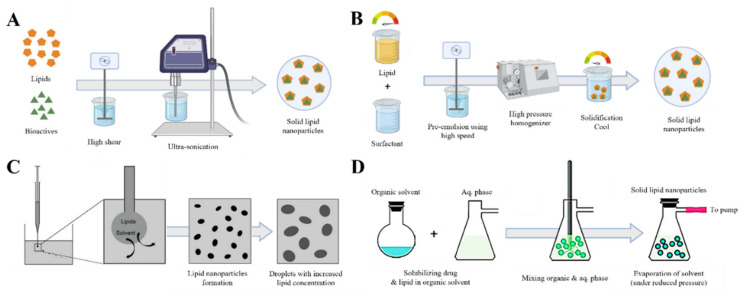
Examples of various LNP emulsification methods. (**A**) Ultra-sonication method. (**B**) High-pressure homogenizer method. (**C**) Solvent injection method. (**D**) Solvent evaporation method. Reproduced with permission from [39] published by Elsevier, 2017, and [37,40] published by MDPI, 2020, 2021.

LNP forms are determined in various ways depending on the material used and the manufacturing method. Traditionally, various LNPs have been synthesized by emulsifying aqueous and organic phases in the presence of a surfactant through machine-based methods (such as a high-pressure homogenizer and ultra-sonication) or solvent-based methods (solvent extraction and evaporation) (Figure 2A–D) [37,38,39,40,41]. There are many perspectives to classify LNPs; however, this review described LNPs based on their core types.

The nano-emulsions are an early LNP model of a liquid core based on liquid lipids, subdivided into W/O, O/W, W/O/W, and O/W/O nano-emulsions based on materials and synthesis methods. O/W and W/O/W are mainly used for administering drugs in an aqueous environment, such as the blood, owing to their hydrophilic surface properties (Figure 1) [42,43]. O/W nano-emulsions (micelle form) have a hydrophobic interior composed of oil; therefore, they are mainly used to deliver lipophilic drugs. W/O/W nano-emulsions have a water phase surrounded by an inner membrane; they are mainly used to deliver hydrophilic drugs but can co-deliver lipophilic drugs because of their organic phase [44,45]. The nano-emulsion types are easier to synthesize than other LNPs. An efficient manufacturing method using self-assembly based on a microfluidic chip for W/O/W nano-emulsion synthesis has been proposed recently [46]. However, high-concentration surfactants are required for manufacturing. Helper lipids (such as cholesterol) must be used to overcome the structural instability of the membrane caused by the liquid-based internal structure of nano-emulsions [24,47,48].

**Figure 3:**
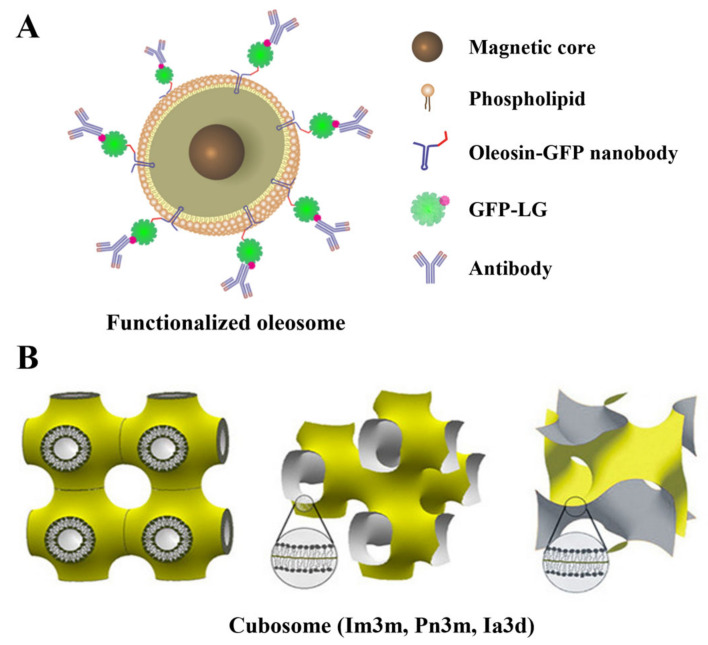
Newly proposed lipid nanoparticles-based drug delivery systems: (**A**) oleosome; (**B**) cubosome. Reproduced with permission from [50,54] and published by the American Chemical Society, 2015, 2018.

Natural nano-emulsions have been proposed as a new DDS for lipophilic drugs. Recently, a natural O/W nano-emulsion called an oleosome was suggested as the delivery vehicle for lipophilic drugs (Figure 3A) [49,50]. Oleosomes have a simple structure: a triacylglycerol core, a component of animal fats/vegetable oils, is surrounded by a single phospholipid membrane and several unique membrane proteins [51,52]. Among these proteins, oleosin improves the structural stability of oleosomes [53]. These structural characteristics suggest using oleosomes as a new DDS. However, few studies have been conducted to date. Abdelalim et al. [49] reported that nano-size oleosomes improve the transdermal drug delivery efficacy of sildenafil citrate and palmar–plantar erythrodysesthesia. Cho et al. [50] used nano-oleosomes as a delivery vehicle for targeted anticancer treatment for the lipophilic drug carmustine.

### 2.2. Solid Lipid-Core LNP (Solid Lipid Nanoparticle)

Solid lipid nanoparticles (SLNs) are the most widely studied LNP for lipophilic drug delivery (Figure 1). SLNs are synthesized through emulsification between an organic phase containing a solid lipid and a water phase containing a surfactant at a temperature slightly higher than the melting temperature of the solid lipid. SLNs consist of a solid lipid internal structure to enhance the stability of the DDS. Therefore, they can protect drugs more from external environmental conditions than nano-emulsion-type LNPs [55]. Therefore, they are attracting attention for oral administration because they can protect drugs from extreme conditions such as gastric acid [26,56]. From a formulation point of view, SLNs offer advantages, such as excellent biodegradability, physical stability, and controlled drug release [57,58,59]. Owing to these properties, SLNs are considered an attractive alternative to emulsion LNPs. Lipophilic drug delivery studies using SLNs have been actively conducted in various disease models (Table 1). However, the high-temperature emulsification process to obtain a uniform solid lipid core can limit using SLNs in delivering drugs unstable to heat and physical shock. In addition, SLNs have limited commercialization because of their high initial burst drug release and leakage during storage owing to the crystallization of solid lipids during the cooling process after synthesis [59,60,61,62].

### 2.3. Nanostructured Lipid Carrier (NLC)

Based on the advantages of the aforementioned LNP types, nanostructured lipid carriers (NLCs) with cores hybridized with liquid and solid lipids have been developed to improve the lipophilic drug delivery efficiency (Figure 1) [60]. NLCs are formed by adding liquid lipids to the high-temperature organic phase during the manufacturing process of SLNs, with oil plots composed of liquid lipids collected inside the solid core during the emulsification process. The formed oil plot induces an increased number of defects in the core–solid matrix, which improves the capture rate by promoting drug inflow into the interior while maintaining the physical stability of the LNCs and preventing drug leakage from the solid lipid-core LNP crystallization [61]. The drug-loading capacity of NLCs can be increased by combining solid and liquid lipids with various properties. Owing to these advantages, NLCs have attracted attention as a DDS (Table 1); however, some unresolved challenges remain. NLCs are synthesized using several lipids; therefore, quality challenges, such as sterilization stability and polymorphic changes in the lipids, may occur [103]. Cytotoxicity due to the nature and concentration of some lipids and surfactants (such as hydrophobic surfactants) used in NLCs synthesis may occur [26]. In addition, the synthesis of NLCs is similar to that of SLNs and thus it was initially considered that NLCs could be used only for delivering heat-stable drugs. However, Gainza et al. [104] modified the synthesis and revealed that NLCs could also be used to deliver heat-sensitive drugs (such as proteins).

### 2.4. Hollow LNPs

Hollow-core LNPs, such as cubosomes, have been proposed as a novel DDS (Figure 3B). They are synthesized from amphiphilic lipids with unique structural properties, such as monoolein [105,106]. These lipids form self-assembled thermodynamically stabilized bicontinuous cubic phases at specific temperatures and phase ratios [107]. After adding a stabilizer (such as a block copolymer), the cubic phase is stabilized by the polymer-based external corona formed during ultrasonication to form a cubosome [108]. Cubosomes have various forms depending on the surfactants ratio and synthesis methods [107,109]. These cubic LNPs have a hollow 3D network structure, and their shells are composed of a surfactant-based bilayer (Figure 3B). Based on these structural characteristics, cubosomes can co-deliver lipophilic (in shells) and hydrophilic (in space) drugs. Cubosomes are highly biocompatible, non-toxic, and thermodynamically stable [110,111,112]. In addition, external polymer-based coronas are stable under physiological conditions and can be used for targeting [108]. These advantages of cubosome suggest that they are ideal drug carriers. However, challenges in large-scale production owing to the high-concentration surfactants, phase behavior, and high-viscosity properties are considered in commercializing cubosomes [112,113].

Over the past few decades, LNPs with various cores have been developed to improve the bioavailability of lipophilic drugs. Many studies on drug protection, efficient capture, and release have focused on changing the internal structure of LNPs based on the materials and methods. However, specific functions, such as targeted delivery, membrane penetration, bio-adhesion, and evasion from the immune responses, are more closely related to the external membrane [114,115,116]. Therefore, many studies have been conducted to impart various functionalities to LNPs through surface modifications.

## 3. Surface Modifications of Lipid Nanoparticles

**Figure 4:**
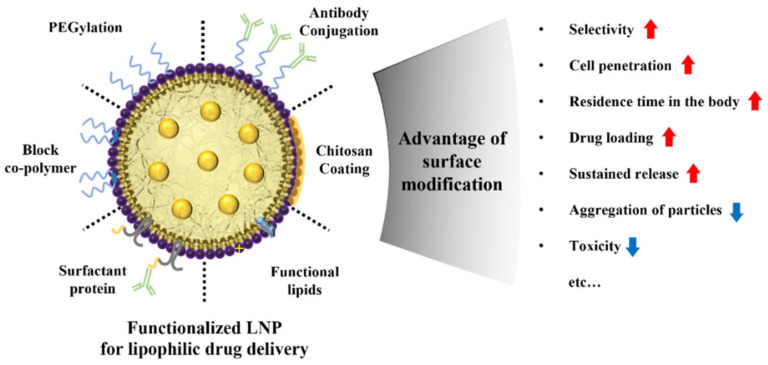
Various surface modifications for high-functionality of lipid nanoparticles.

Surface modifications involve changing the carrier’s surface in various ways. DDSs use the direct contact between the surface of a carrier and the target to achieve efficient drug delivery [115,116]. These technologies mainly involve loading active agents onto the membrane of a carrier or coating the entire membrane (Figure 4). These modifications impart high functionalities to LNPs, such as a targeting ability, cell membrane penetration, and drug release control. In addition, LNP functions related to stability in the body, including increased residence time and prevention of aggregation, can be improved via surface modification. As described in Table 2, various LNP surface modifications are being applied to improve their bioavailability. Here, the various types of surface modifications applied to LNPs and their functions are discussed.

### 3.1. Polymer

**Figure 5:**
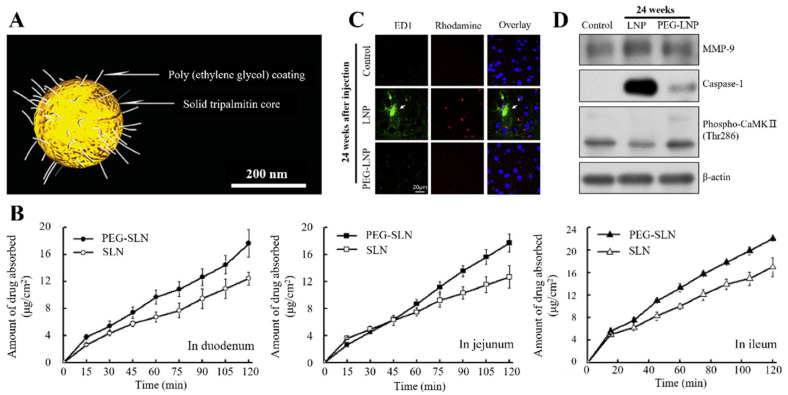
LNP Functionality by polymer-based surface modification. (**A**) LNPs stabilized by PEGylation. (**B**) Mitigation of microglia activation by injecting LNPs through PEGylation. After injecting nanoparticles, the microglia activation was examined via immunostaining (ED1-based). ED1-positive cells with amoeboid morphology are indicated using Arrow (scale bar = 20 μm). (**C**) Mitigation of neurovascular damage by PEGylation. Immunoblot images of mouse brains according to LNP injection probed with anti-MMP-9 (blood–brain barrier integrity marker), caspase-1 (inflammatory signal marker), and phospho-CaMKII (synaptic stimulation marker) antibodies. Anti-β-actin was used as a loading control. (**D**) Absorptive characteristics of SLN and PEGylated SLN in the everted rat gut sac system on three intestines. Reproduced with permission from [139], published by Elsevier, 2013, and [121,122] published by the American Chemical Society, 2004, 2013.

Polymers are the most common LNP surface modifiers. Their unique structures impart special functions to carriers, such as increased residence time in the body and targeting ability [117,118]. PEGylation is a surface modification strategy used to improve the bioavailability of various LNP-based DDSs by modifying polyethylene glycol (PEG)-attached membrane lipids (Figure 5A) [136]. PEG has dissolution properties in water and polymers and is harmless to the human body because it is non-antigenic [137]. The PEGylated carriers become stealth from the reticuloendothelial system (RES), increasing persistence in the body and inducing the enhanced permeability and retention (EPR) effect, which imparts the passive targeting ability of tumor cells [119,120]. Liu et al. [32] reported that the amphiphilicity of PEG could increase the drug release rate by reducing the surface tension of LNPs. Moreover, LNP surface modification using PEGylated lipids forms a polymer layer on the exterior membrane to improve the membrane stability and prevent nanoparticle aggregation [121,138].

Various DDS studies have demonstrated the enhanced drug delivery efficiency by PEGylation in LNPs. Yuan et al. [122] evaluated the bioavailability enhancement of PEGylation after the oral administration of SLNs. According to this study, PEGylated-SLNs (PEG-SLNs) increase penetration into the mimetic intestinal epithelial cell (Caco-2/HT29) monolayer and displays a higher stability in simulated intestinal fluid (Figure 5B). In addition, pharmacokinetic studies have revealed that SLN bioavailability changes approximately two-fold via PEGylation. In PEGylated LNPs (PEG-LNPs), the oral absorption rate of fenofibrate is increased. Furthermore, simulated lipolysis in digestive fluid has suggested that PEGylation resists the degradation of LNPs from lipase [140]. Additionally, the stability of PEG-LNPs is maintained even under bile acid exposure conditions [141].

The notable functional improvement via LNP PEGlyation has also been studied in injectable formulations. PEGylated-NLCs (PEG-NLCs) were used as an injection preparation loaded with baicalin, a lipophilic drug that improves the cardiovascular function. The PEG-NLCs displays a three-fold longer residence time in the body than normal NLCs in pharmacokinetic analysis [142]. These results suggest that PEG reduces reticuloendothelial immune activity against NLC and prevents aggregation with plasma proteins [84,143]. Along with the EPR effect, PEG may enhance anticancer drug delivery by accumulating LNPs in tumor cells [117]. In addition, PEG-LNPs improve biodistribution by inhibiting transporter aggregation and mitigating microglial activation and neurovascular damage related to the immune response in the brain (Figure 5C,D) [139].

Based on the high targeting ability of PEG-LNPs, Abdel Fadeel et al. [68] studied the drug delivery efficiency of the skin anticancer drug curcumin prepared as PEG-SLNs. The drug delivery rate through PEG-SLNs was over twice that of the suspension, attributed to PEG-LNPs, improving the penetration and accumulation rate of the drug in the skin layer. Dang et al. [144] demonstrated the effective delivery of latanoprost in ocular DDS by improving the transmittance and swelling of the contact lens owing to the higher adhesion of the protein with PEG-SLNs. The suspension-based lens released all the drugs in 24 h; nonetheless, PEG-SLNs maximally increased the duration to 96 h. Animal studies have revealed that PEG-SLNs have fewer side effects in vivo, suggesting that they can be an alternative to eye drops for drug delivery through contact lenses.

Studies are being conducted to impart a better targeting ability to LNPs by conjugating antibodies to a carrier’s surface using PEG lipids with modified terminals. Liu et al. [145] conducted a study on loading baicalin into SLN PEGlyated with PEG-maleimide (MAL). This modified PEG lipid was combined with an OX26 antibody specific to thiolated brain cells through a maleimide–thiol reaction [146]. The targeting ability of the cells was improved after conjugation. This conjugation reaction also contributed to the emergence of PEGylated cubosome-based DDSs specific for the epidermal growth factor receptor (EGFR) [147]. In a study by Kebebe et al. [118], carboxyl acid-exposed PEG-NLCs and tumor-targeting peptides were conjugated using the EDC-NHS reaction. They developed effective breast cancer-targeting NLCs. Li et al. [148] conjugated TLNk, a protein with keratinocyte targeting ability, using PEG-amine and BS (PEG)_5_ (homobifunctional crosslinker) to LNPs. The modified LNPs had higher skin regeneration rates compared to the LNPs in the burnt skin of mice by the targeting ability.

In addition to PEGylation, block copolymers (BCPs) based on polyethylene oxides and polypropylene oxides, such as poloxamers (Pluronic^®^) and poloxamines (Tetronic^®^), have been utilized for LNP surface modification. They are excipients in various drugs owing to their adsorption and association properties derived from their unique structure-based amphiphilic properties and low cytotoxicity [149,150]. Among these excipients, BCPs help improve the LNP bioavailability, enhance the carrier stability, and increase the residence time in the body, similar to PEGylation [123,124,151,152].

Studies have suggested that BCPs, especially poloxamer surface-modified LNPs, increase the cellular uptake and targeting ability of delivery vehicles through interaction with plasma proteins. Göppert and Müller [153] compared the adhesion rates of various apolipoproteins (apo) with SLNs stabilized with low molecular weight poloxamers (184 and 235). This confirms the high adsorption of apo E, which mediates uptake across the blood–brain barrier (BBB). Poloxamer 235 demonstrated a dramatically low adsorption rate for apo CII, which inhibited the apo E receptor function. In addition, the unimer of poloxamer 235 inhibits the glycoprotein P efflux pump of bovine brain endothelial cells, resulting in an increased drug accumulation in the cells [125]. Therefore, surface-modified SLNs with poloxamer 235 could be the most suitable vehicle for brain-targeting DDSs [153].

Currently, research results have been reported to improve the bioavailability of LNPs through surface modification based on special polymers. Cationic polyelectrolyte poly (allylamine hydrochloride) not only reduces MnO_4_ to MnO_2_ during LNP fabrication but also acts as a protective layer to stabilize LNPs due to electrostatic repulsion. However, the polyelectrolyte-coated MnO_2_-LNPs cause instability and cytotoxicity in cell media or saline due to their small particle sizes and positively charged properties [154]. Gordijo et al. [155] synthesized two hybrid LNPs, inserting polyelectrolyte-MnO_2_ (PMD)-LNPs into hydrophilic terpolymer/protein-MnO_2_ (TMD) or hydrophobic polymer/lipid-MnO_2_ (LMD) matrices in order to improve the hypoxia of the tumor microenvironment (TME) and prevent colloidal MnO_2_ from reacting rapidly with H_2_O_2_. Compared to PMD-LNPs, the prepared hybrid LNPs had significantly improved the colloidal properties and biocompatibility both in vitro and in vivo, and they were non-toxic to tissue during one week of intravenous (IV) administration. LMD-LNPs effectively reduced hypoxia and HIF-1α in solid tumors by their lower reactivity and excellent tumor accumulation and retention at a normal pH level (pH 7.4). In addition, hydrophilic TMD-LNPs with a cluster structure generate oxygen faster than denser hydrophobic LMD-LNPs, making them suitable for intratumoral injection.

Wang and his team [156] studied layer-by-layer coated SLNs composed of natural biopolymers, such as sodium caseinate (NaCas) and pectin layers. Amphiphilic NaCas can be adsorbed and emulsified at the interface between oil and water [157], and pectin forms complex particles to stabilize NaCas particles at an acidic pH [158]. The particle size increased after the pectin coating process, indicating that pectin was adsorbed on the NaCas-SLN surface. All pectin-coated NaCas-SLNs showed PDI values less than 0.3 and were operated normally by drug loading. Thus, the aforementioned results indicate that the pectin coating improved the stability and encapsulation function of NaCas-SLNs.

### 3.2. Chitosan Coating

The biopolymer-based surface coating has been proposed as a new surface modification method to improve LNP bioavailability. LNPs coated with a layer composed of biopolymers can prevent factors that hinder the stability of LNP colloids, such as interparticle aggregation or polymorphic transitions [126,159]. Biopolymer-based coatings are primarily formed by electrostatic interactions between the negatively charged carboxyl group of lipids and positively charged polymers [160].

Chitosan, a natural cationic polysaccharide derived from chitin, is a representative biopolymer used in LNP surface modification. Chitosan has a low cytotoxicity and excellent biocompatibility and biodegradability [160]. In addition, it has been widely used in the pharmaceutical field owing to its wound-healing activity, antibacterial activity, and excellent bioadhesive properties on negatively charged surfaces, such as the skin and mucous membranes [161,162]. Therefore, LNPs coated with chitosan can easily penetrate the mucosal barrier and exhibit extended retention in the body, thereby improving the LNP bioavailability [83,163,164].

Chitosan coatings are usually applied to LNPs, particularly SNLs and NLCs. Luo et al. [126] used chitosan-coated SLNs (CS-SLNs) for the oral delivery of the lipophilic drug coumarin-6. They confirmed that chitosan improves the SLN stability in acidic environments. They compared and analyzed the difference in the membrane potential between SLNs and CS-SLNs by measuring the zeta potential to confirm that SLNs were coated with chitosan. The membrane potentials of their synthesized SLNs and CS-SLNs were measured as negative-to-positive charge changes to −15.9 mV and 26.1 mV.

**Figure 6:**
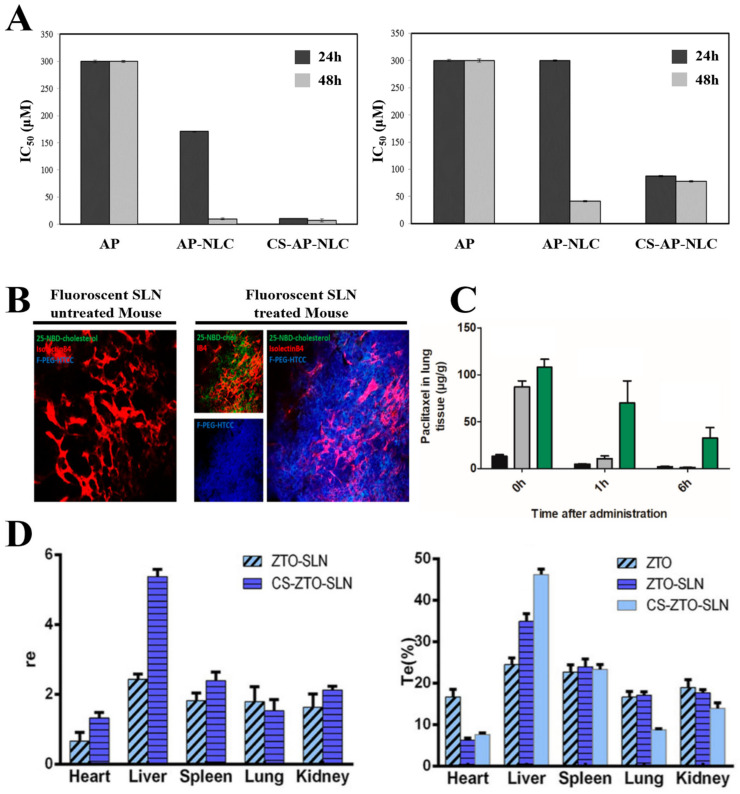
Functionalization of LNPs through chitosan coating. (**A**) In vitro dose-dependent NLC and CS-NLC cytotoxicity profiles on (**left**) Caco-2 and (**right**) Hela cells. (**B**) In vivo tumor distribution of fluorescent-CS-SLN loaded with 25-NBD-cholesterol following inhalation on the M109 model. Confocal images of fluorescent SLN untreated or treated M109 mouse lung. (Red: vessels labeled with isolectinB4), (green: 25-NBD-cholesterol labeling SLN), (blue: Alexa Fluor 405-grafted-F-PEG-HTCC labeling the coating). (**C**) Pulmonary exposure to paclitaxel after administering intravenous (black: Taxol), inhaled (gray: Taxol), and inhaled (green: F-PEG-HTCC-coated SLN). (**D**) The evaluation of chitosan coating’s targeting efficiency in mouse organs, including the heart, liver, spleen, lung, and kidney. The relative uptake ratio (**left**), targeting efficiency (**right**). CS: chitosan coating. Reproduced with permission from [83,127] published by Elsevier, 2015, 2017, 2020, and [130] published by the American Chemical Society, 2018.

Yostawonkul et al. [127] used chitosan-coated NLCs (CS-NLCs) for the oral administration of the anticancer drug alpha-mangostin (AP). A comparison of free AP, NLC, and CS-NLC against carcinoma Caco-2 cells and HeLa cells revealed the highest anticancer activity in CS-NLCs (Figure 6A). This was presumed to be owing to the high mucoadhesive properties caused by electrostatic interactions between the CS-NLCs and cell membranes [83]. Additionally, cytotoxicity tests on zebrafish embryos revealed an improved cellular uptake in CS-NLCs compared with surface-unmodified NLCs. However, as the toxic effects of CS-NLC on the cells increased, the research team mentioned that it is necessary to set the optimal concentration without toxicity when using CS-NLCs.

Gartziandia et al. [89] confirmed the CS-NLC-based drug delivery efficiency through the nasal cavity. They reported that chitosan has an excellent adsorption ability to nasal epithelial cells through NLC. Electrostatic interactions between chitosan and epithelial cells reduce the disintegration of transporters in the nasal epithelium [165]. Additionally, chitosan absorbs water in the mucous layer of the nasal cavity and swells to form a gel layer, thereby extending the residence time of the delivery system [166]. In this study, CS-NLCs did not cause red blood cells agglutination or toxic reactions in the nasal mucosa of mice.

Chitosan-coated LNPs have a higher corneal permeability for ocular drug delivery. Wang et al. [128] compared the delivery efficiency of the lipophilic drug methazolamide to CS-SLNs and SLNs in rabbit corneal cells. CS-SLNs displayed a higher permeability into corneal cells, and no eye irritation was observed. In addition, chitosan coating can improve the physical stability and extend the storage period of SLNs by over two times at 4 °C. Sohaib et al. [129] confirmed that the drug-loading rate, drug release duration, and skin penetration were improved when finasteride, a lipophilic drug, was delivered to the skin through CS-SLNs in animal experiments.

Rosiere et al. [130] reported that while inhaling and delivering paclitaxel, a lipophilic drug for lung cancer treatment, CS-SLNs increased the drug residence time in the lungs and improved intracellular penetration (Figure 6B,C). They used N-[(2-hydroxy-3-trimethylammonium)propyl] chitosan chloride (HTCC), a chitosan derivative with a low molecular weight, because the strong positive charge on the surface of the carrier generated using high molecular weight chitosan could cause cytotoxicity. They conjugated folate-PEG-COOH with HTCC in SLNs, imparting a targeting ability to cancer cells with more folate receptors on the membrane than normal cells [167]. Therefore, modified CS-SLNs could continuously deliver drugs to the lung tissue for up to 6 h in mouse experiments. Yang et al. [83] used CS-SLNs in experiments with mice to improve the low bioavailability of antitumor-related lipophilic drug zedoary turmeric oil (ZTO) against liver cancer. According to tissue homogenate HPLC analysis, the drug significantly accumulated in the liver (Figure 6D).

### 3.3. Functional Lipid

Specific lipids can impart various functions to LNPs through surface modifications. Cholesterol has been principally used as a helper lipid to increase LNP membrane stability [168,169]. Studies have suggested that cholesterol confers other specialized functions to LNPs in addition to the stability. The high cholesterol contents of LNP increase the BBB permeability [77] and hepatocyte uptake of lipid nanoparticles [131]. Furthermore, cholesterol provides lipid nanoparticles with an improved drug-loading capacity [132].

During intracellular drug delivery, endosome escape is critical for determining the drug delivery efficiency. Phytosterol (β-sitosterol), a component of plant cell walls and a cholesterol analog, enhances LNPs escape from endosomes [33,133]. Thus, adding β-sitosterol to the LNP membrane forms more angular parts, which induces binding to the endosome membrane and provides LNPs with a higher ability to escape endosomes. In addition, β-sitosterol-based LNPs demonstrated an endosome escape ability over 10 times that of cholesterol in live-cell imaging analysis based on the Gal8-GFP reporter system [133].

In contrast, ionizable cationic lipids have also induced the endosomal escape of LNPs in response to pH composition. In the case of a general intravenous administration, lipids such as 1,2-dioleoyl-3-dimethylammonium-propane; DODAP have a near-neutral surface charge at a neutral pH but change to a strong positive charge at an acidic pH [134]. Endosomes that trap intracellular LNPs are transformed into lysosomes with an acidic pH by fusion with vesicles containing lysosomal hydrolases transferred from the trans-Golgi network. This lipid exhibits a strong positive charge and induces an unstable LNP structure formation, which induces LNPs escape from endosomes [148,170,171].

Recently, studies have been conducted on the efficiency of charge neutralization in LNP membranes using cationic lipids. Cationic lipids have been principally used to trap nucleic acids, which are hydrophilic drugs, in the hydrophobic LNP interior via ion pairing [24]. Several cationic lipid-based surface modification studies have suggested that the bioavailability of LNPs could be improved. Cationic-LNPs exhibit a high cellular uptake and improved drug-loading capacity through electrostatic interactions [70,172,173,174]. Sood et al. [78] reported that C-SLNs synthesized through microemulsion technology improves the oral bioavailability of a lipophilic antipsychotic drug (olanzapine) over four times in mouse experiments. They suggested that a direct absorption from the gastrointestinal tract and avoidance of the hepatic metabolism through C-LNPs contributed to the improved bioavailability of the drug. Liu et al. [175] also reported that C-SLNs more than doubled the gastrointestinal absorption of N3-O-toluyl-fluorouracil (TFu), a lipophilic anticancer drug. Another study on C-SLNs reported that cationic lipids could reduce the clearance interference via immune cells by neutralizing the negative charge of the LNP membrane [148]. Despite the various advantages of these cationic lipids, there have been ongoing discussions about their cytotoxicity [176,177]. Therefore, to continuously lower the cytotoxicity of delivery systems using cationic lipids, alternative methods, such as developing two-tailed cationic lipids with a lower cytotoxicity than existing ones, have been proposed [178,179].

### 3.4. Surfactant Protein

**Figure 7:**
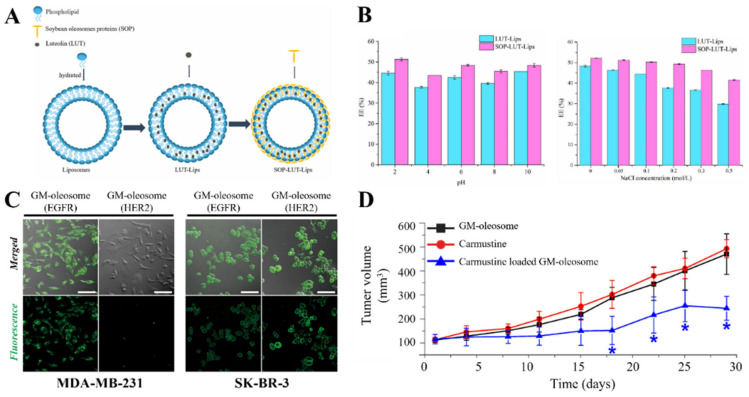
Functionalization of liposome and LNPs coated with surfactant protein. (**A**) Schematic illustration of the formation of luteolin-loaded liposomes coated with soybean oleosin (SOP-LUT-Lips). (**B**) Encapsulation efficiency of LUT-Lip and SOP-LUT-Lip under different pH conditions and ionic strength conditions. (**C**) Selectivity test of GM-oleosome. EGFR is not specific for either breast cancer cell line. However, HER2 is selective for SK-BR-3. Observation was conducted via confocal microscopy. Scale bar is 100 μm. (**D**) Increased carmustine delivery capacity of GM-oleosome (* *p* < 0.001 compared to G2 and G3). Reproduced with permission from [135], published by Elsevier, 2022, and [50] published by the American Chemical Society, 2018.

Oleosin, which is a representative surfactant protein, has a unique amphipathic topological structure and enhanced membrane stability. Thus, studies are being conducted on surface modification using oleosin to impart stability and functionality to the liposomes and LNPs. Li et al. [135] proposed liposomes coated with soybean oleosin as a drug delivery platform for the lipophilic drug luteolin (Figure 7A). In the aforementioned study, oleosin improved the structural stability of the liposome membrane and enhanced resistance to various environmental stresses (such as ion, pH, and temperature) (Figure 7B). Cho et al. [50] used LNPs coated with genetically modified oleosin (GM-oleosome) hybridized with a GFP-nanobody for the targeted delivery of a lipophilic anticancer drug (carmustine) to breast cancer. They developed a highly functional LNP capable of targeting and tracking by combining the GFP-LG protein and HER2 antibody (specific to SK-BR-3 cells) to this carrier without chemical treatment-based conjugation (Figure 3A). In vivo tests with a mouse confirmed that the GM-oleosome selectively delivered drugs to target cancer cells (Figure 7C,D). Overall, the aforementioned results suggest that natural surfactant proteins, such as oleosin, are effective surface modifiers for manufacturing high-functionality LNPs.

## 4. Conclusions

LNPs are becoming a central DDS platform for delivering lipophilic drugs with a low water solubility. Over the past decades, LNPs have been subdivided into different materials and synthetic methods depending on the desired purpose. Among them, LNPs with various core types, ranging from traditional nano-emulsions with liquid cores to cubosomes with a hollow core, have been developed to improve the bioavailability of lipophilic drugs. Through these efforts, LNPs have overcome the existing limitations of DDSs and proved to be the most suitable for lipophilic drug delivery.

LNP-based therapeutics, including various lipophilic drugs, are being developed for treating diseases. However, to commercialize them as ideal DDS carriers, many challenging tasks, such as the targeting ability, cell penetration, and endosome escape, remain. To solve these problems, the complete application of surface modification, which provides a special function by modifying the membrane of the carrier, has been proposed. Various surface modifications have been developed to make LNPs highly functional, ranging from PEGylation, which is most commonly used in LNP-based DDS, to surfactant proteins that have recently emerged. Various surface modifiers impart stability and targeting ability to LNPs to minimize side effects and hide them from the immune system, resulting in a longer residence time in the body. In addition, surface modifications using materials with special functions, such as bio-adhesives and endosome escape, increase the LNP absorption rate in vivo.

In conclusion, a number of recent LNPs studies suggest that LNPs and surface modifications can significantly improve the bioavailability of lipophilic drugs. However, the potential side-effects, such as allergic reactions to LNPs and the difficulty of scale-up of most surface modifications, remain a major obstacle to their commercialization. If these problems are solved through many clinical studies of LNPs and optimization of the surface modification process, cooperation between LNPs and surface modification can play an important role in making LNPs the most suitable DDS for lipophilic drug.

## Figures and Tables

**Table 1:** Recent lipophilic drug delivery based on various types of lipid nanoparticles.

Type of LNPs	Target	Drug	Lipid and Surfactant	Therapeutic Effect	Reference
Nano emulsion (liquid-core LNPs)	Eye	Dexamethasone	Isopropyl myristate, Tween 80, propylene glycol	Treatment of acute and chronic eye disease such as uveitis	[63]
	Eye	Everolimus	Triacetin, poloxamer 184, propylene glycol	Immunosuppressive drug to prevent corneal graft rejection	[64]
	Brain	Indinavir	Soybean oil, Tween 80, EPC-80 (egg yolk lecithin), oleic acid, α-tocopherol	Treatment of human immunodeficiency virus (HIV) infection	[65]
	Brain	Saquinavir mesylate	Capmul MCM, Tween 80, PEG 400, isopropyl myristate	Treatment of human immunodeficiency virus (HIV) infection	[66]
	Brain	Risperidone	Capmul MCM, Tween 80, transcutol, propylene glycol	Antipsychotic drug	[67]
	Skin	Curcumin	Tefose 1500 mixed PEG-6 stearate and PEG-32 stearate), Span 85, Span 20, Tween 80, Tween 20	Targeted therapies for skin cancer	[68]
	Breast	Carmustine	Olive oil, 1,2-dioleoyl-sn-glycero-3-phosphocholine (phospholipid)	Targeted therapies for breast cancer	[50]
SLN	Eye	Atorvastatin	Compritol^®^ 888 ATO, Phospholipon 90 H, poloxamer 188, PEG 400	Treatment of age-related macular degeneration	[69]
	Eye	Melatonin	Stearic or palmitic acid, cationic lipid, Didecyldimethylammonium bromide, Softisan 100, Tween 80	Increase the ocular hypotensive effect of drugs and treat anti-glaucoma	[70]
	Eye	Indomethacin	Compritol ATO 888, Tween 80, poloxamer 188, glycerin	Treatment of posterior segment of eye disease	[71]
	Eye	Diclofenac	Compritol 888 ATO, Precirol 5 ATO, hydrogenated soy PC, poloxamer 188	Improve analgesic and anti-inflammatory drug toxicity	[72]
	Eye	Idebenone	Stearic acid or palmitic acid, Softisan 100, Tween 80, didecyldimethylammonium bromide	Leber’s hereditary optic neuropathy	[73]
	Brain	Docetaxel, ketoconazole	Glyceryl monostearate, soy lecithin, vitamin E, Tween 80	Brain-targeted anticancer drug that penetrates the blood–brain barrier	[74]
	Brain	Apolipoprotein E-derived peptide	Dynasan 116, Epikuron 200	Penetrate the blood–brain barrier (BBB)	[75]
	Brain	β-elemene	Glyceryl monostearate, glycerol tristearate, sodium cholate	Blood–brain barrier penetration and neurotherapy	[76]
	Brain	Saquinavir	Cacao butter, cholesterol, stearylamine, esterquat 1, Tween 80	treatment of human immunodeficiency virus (HIV) infection	[77]
	Brain	Olanzapine	Stearic acid or glyceryl monostearate, soy lecithin, poloxamer 188, stearyl amine	A psychotropic agent that belongs to the thienobenzodiazepine class and is indicated for acute and maintenance treatment of schizophrenia	[78]
	Skin	Naproxen	Glyceryl monostearate, Span 80, Tween 80	Reduce side effects of systemic absorption of drugs and increase drug concentration at the site of action/treatment of rheumatic diseases and related pain conditions	[79]
	Skin	Adapalene	Steric acid, trimyristin, glyceryl monostearate, glyceryl monooleate, Compritol 888 ATO, Precirol ATO 5, Brij 78, Pluronic F68, Tween 80, Span 20	Treatment of acne	[80]
	Skin	Spironolactone	Stearic acid, Tween 80, Span 80, Span 60	Treatment of skin disorders	[81]
	Skin	Resveratrol	Stearic acid, soy phosphatidylcholine, poloxamer 407	Treatment of skin disorder	[82]
	Liver	Zedoary turmeric oil	Glycerin monostearate, glycerol, Tween 80	Strong antitumor activity	[83]
	Lung	Artemether	Glyceryl monostearate, Compritol 888 ATO, stearyl amine, MPEG2000-DSPE, Cremophor EL, poloxamer 188, poloxamer 407, Solutol HS	Improve oral bioavailability and treat lung cancer	[84]
NLC	Eye	Triamcinolone acetonide	Precirol^®^ ATO 5, Squalene^®^, Lutrol^®^ F68, Monoolein	Treatment of posterior segment diseases	[85]
	Eye	Dexamethasone	Labrafac™ lipophile WL1349, Tween 80, Cholesterol	Treatment of dry eye disease (DED) or keratoconjunctivitis sicca	[86]
	Eye	Itraconazole	Tripalmitin, transcutol HP Chitosan, Tween 80	Anti-neovascularization effect and treatment of diabetic retinopathy (DR)	[87]
	Eye	Propranolol hydrochloride	Compritol ATO 888, oleic acid, Transcutol P, Tween 80, Span 20	Treatment of posterior segment of the eye disease	[88]
	Brain	Insulin	Precirol ATO5, Miglyol, Tween 80, poloxamer 188	Penetrate the blood–brain barrier to treat the central nervous system	[89]
	Brain	Atazanavir	Precirol ATO5, Lauroglycol 90, Cremophor RH 40	Treatment of neuro-AIDS	[90]
	Brain	Vinpocetine	Compritol 888 ATO, Monostearin, Miglyol 812N, Solutol HS-15 or poloxamer 188, lecithin	Treatment of chronic cerebral vascular ischemia, acute stroke, senile cerebral dysfunction, and Alzheimer’s disease	[91]
	Brain	Olanzapine	Glyceryl tripalmitate, castor oil, Pluronic F-68, soy lecithin	Treatment of schizophrenia	[92]
	Liver	Adefovir dipivoxil	Precirol ATO5, Capmul MCM, Cremophor RH 40, poloxamer 188, egg yolk lecithin	Treatment of hepatitis B virus infection	[93]
	Liver	Atorvastatin	Gelucire^®^ 43/01, Compritol^®^ 888 ATO, Capryol^®^ PGMC, Pluronic^®^ F68, Tween^®^ 80	Decrement of cholesterol and triglyceride (fats) levels in the blood	[94]
	Liver	Naringenin	Stearic acid, monostearin, oleic acid, poloxamer 188, soybean lecithin	Inhibition of nonalcoholic fatty liver disease	[95]
Cubosome	Peritoneal macrophage	Antigen, Polysaccharide	Phytantriol, propylene glycol, Pluronic F127	Increase the ability of immunostimulants	[96]
	antidiabetic activity	Gliclazide	Glyceryl monooleate, poloxamer 407	Improve antidiabetic activity	[97]
	Cell	Elesclomol copper complex	Monoolein, poloxamer 407 (PF127)	Anticancer drug for skin cancer, intractable solid cancer, and blood cancer	[98]
	Eye	Voriconazole	Monoolein, Pluronic F127	Treatment of fungal keratitis	[99]
	Brain	Curcumin	Monoolein, fish oil, PEG1000	Treatment of neurodegenerative disease	[100]
	Brain	Piperine	Glyceryl monooleate, Tween 80, poloxamer 407, Cremophor	Treatment of Alzheimer’s disease	[101]
	Skin	Paclitaxel	Monoolein, DSPE-PEG-ma, Pluronic F127	Treatment of skin cancer	[102]

**Table 2:** Surface modification types and functions applied to LNPs.

Surface Modifier	Function	Reference
PEGylation	- Increase the stability	[117] [118] [119] [120] [121] [122]
	- Increase the residence time in the body	
	- Increase drug stability	
	- Increase the absorption rate for oral administration	
	- Increase drug penetration and accumulation rate in cells	
	- Increase resistance to digestive enzymes	
	- Increase drug-loading capacity	
	- Drug release control	
	- Decrease particles aggregation	
	- Decrease immunogenicity by stealthing LNPs from reticuloendothelial system (RES)	
	- Based on the EPR effect, it imparts (passive targeting ability) to LNPs for tumor cells	
	- Targeting ability can be imparted to LNPs through antibody conjugation (based on chemical treatment)	
Block co-polymer	- Increase the stability	[123] [124] [125]
	- Increase the residence time in the body	
	- Increase the cellular uptake and targeting ability	
	- Increases the adsorption rate for apoE, which increases the uptake rate of LNPs in the brain	
Chitosan coating	- Increase the stability of LNPs (especially in acidic environment)	[83] [89] [126] [127] [128] [129] [130]
	- Increase the residence time in the body	
	- Increase the absorption rate for oral administration	
	- Increase mucosal adhesion	
	- Increase delivery to the lungs via inhalation	
	- Increase drug delivery to brain	
	- Increase permeability to corneal cells	
	- Increase skin penetration	
	- Increase intracellular penetration	
	- Increase sustained release time	
	- Increase drug-loading capacity	
	- By positively charging the membranes of LNPs, allowing higher contact with cells that have negatively charged membranes	
Functional lipid	- Increase the stability	[33] [77] [78] [131] [132] [133] [134]
	- Increase the residence time in the body	
	- Increase drug delivery to brain (cholesterol)	
	- Increase uptake by hepatocytes (cholesterol)	
	- Increase drug-loading capacity (cationic lipid)	
	- Increase endosome escape ability (β-sitosterol)	
	- Decrease clearance interference by immune cells (through neutralizing the negative charge of the LNP membrane) (cationic lipid)	
Surfactant protein	- Increase the structural stability of the membrane	[50] [135]
	- Increase resistance to various environmental stress (i.e., ion, pH, and temperature)	
	- Antibody conjugation for increasing targeting ability of LNPs based on non-chemical treatment through genetic modification	

## Data Availability

Data sharing not applicable to this article.
